# Emerging Role of TCA Cycle-Related Enzymes in Human Diseases

**DOI:** 10.3390/ijms222313057

**Published:** 2021-12-02

**Authors:** Woojin Kang, Miki Suzuki, Takako Saito, Kenji Miyado

**Affiliations:** 1 Department of Reproductive Biology, National Research Institute for Child Health and Development, Tokyo 157-8535, Japan; sb14536z@st.kitasato-u.ac.jp (M.S.); miyado-k@ncchd.go.jp (K.M.); 2 Department of Applied Life Sciences, Faculty of Agriculture, Shizuoka University, Shizuoka 422-8529, Japan; saito.takako@shizuoka.ac.jp

**Keywords:** TCA cycle, human diseases, TCA cycle-related enzymes, calcium oscillations, mitochondria

## Abstract

The tricarboxylic acid (TCA) cycle is the main source of cellular energy and participates in many metabolic pathways in cells. Recent reports indicate that dysfunction of TCA cycle-related enzymes causes human diseases, such as neurometabolic disorders and tumors, have attracted increasing interest in their unexplained roles. The diseases which develop as a consequence of loss or dysfunction of TCA cycle-related enzymes are distinct, suggesting that each enzyme has a unique function. This review aims to provide a comprehensive overview of the relationship between each TCA cycle-related enzyme and human diseases. We also discuss their functions in the context of both mitochondrial and extra-mitochondrial (or cytoplasmic) enzymes.

## 1. Introduction

Mitochondria, the energy powerhouse of the cell, are important organelles involved in ATP production. Mitochondrial metabolism is essential for the retention of the tricarboxylic acid (TCA) cycle function as mitochondrial metabolism is closely connected with the TCA cycle [1].

In addition to energy production through oxidative phosphorylation, mitochondria are also involved in other important cellular functions, such as homeostasis, defense against oxidative stress, and apoptosis [2]. Mitochondria undergo fusion and fission to maintain normal cell functions [3]. Mitochondria are increasingly recognized as important organelles involved in the aging process. Diseases and aging contribute to disturbances in mitochondrial function; in particular, mitochondrial involvement in neurodegenerative diseases is well known [4].

**Figure 1:**
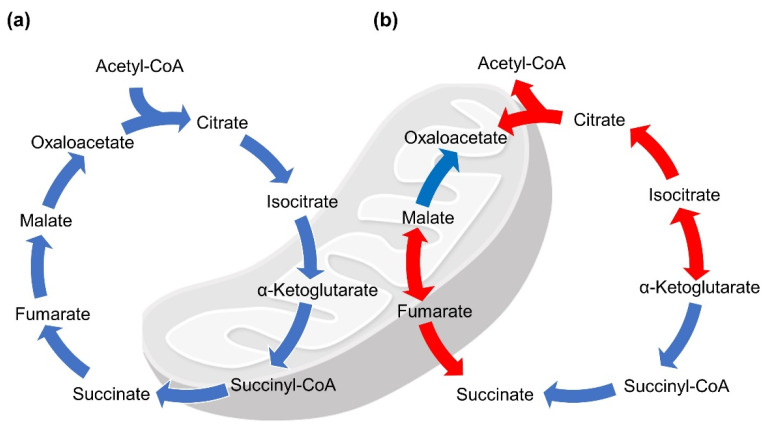
Tricarboxylic acid (TCA) cycle and reverse TCA cycle. Reactions occur in both cycles. (**a**) Well-known classical reactions are shown in blue. (**b**) Reductive and oxidative reactions found in a thermophilic bacterium, *Thermosulfidibacter takaii* ABI70S6^T^, are shown in red.

The TCA cycle, occurring in the mitochondria, generates large amounts of energy under aerobic conditions [1,5]. The TCA cycle oxidizes acetyl-coenzyme A (acetyl-CoA) derived from carbohydrates, fatty acids, amino acids, and ketone bodies, and produces NADH and FADH_2_ for the electron transport chain (Figure 1a). In addition, the TCA cycle provides intermediates that are utilized in the synthesis of glucose, lipids, and amino acids. In this process, eight enzymes oxidize acetyl-CoA and two molecules of carbon dioxide (removed by the reduction of NAD^+^ to NADH) are released [6].

In contrast, there are many anaerobic organisms, such as eubacteria and archaea, that use reverse TCA (rTCA) to produce carbon compounds from carbon dioxide and water [7]. To move reduction reactions using carbon compounds, the rTCA cycle can occur enzymatically and non-enzymatically in both directions, depending on environmental conditions (Figure 1b) [8,9]. Although most TCA cycle-related enzymes are involved in the rTCA reactions, three enzymes, ATP-citrate lyase, 2-oxoglutarate synthase, and fumarate reductase, are known to play a pivotal role [10,11]. In 2018, it was found that citrate synthase (CS) can regulate the reductive and oxidative direction in the rTCA cycle of *Thermosulfidibacter takaii* ABI70S6^T^, suggesting that this reversible enzyme may convert citrate [12].

Some isoforms of TCA cycle-related enzymes are localized in the cytosol, but not in the mitochondria (Table 1). The presence of their activities and refined cycles, integrating several metabolic pathways, such as the metabolism of amino acids and fatty acids in the cytosol, is potentially indicative of functional uniqueness, implying the possibility of novel functions in the extra-mitochondrial space.

**Figure 2:**
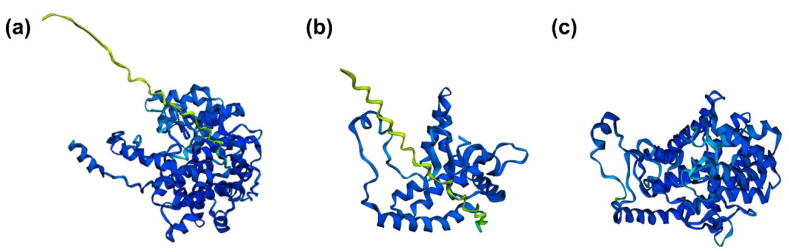
Three-dimentional structures predicted by AlphaFold2. (**a**) citrate synthase (CS), (**b**) extra-mitochondrial CS (eCS), and (**c**) CS (*Thermosulfidibacter takaii* ABI70S6^T^). To perform the structural analysis, amino acid sequences corresponding to CS (GenBank accession no. NP_080820.1), eCS (referred to as “CS-like” in the NCBI database; GenBank accession no. NP_092221.2), and CS (GenBank accession no. BAT71583.1) were analyzed by the simplified version of AlphaFold2 with Colab notebook (https://colab.research.google.com/github/sokrypton/ColabFold/blob/main/AlphaFold2.ipynb, accessed on 26 October 2021). Colored regions indicate confidence of the predicted structure. Dark blue-colored regions indicate high accuracy (>90%) and decrease in accuracy is shown in the order of light blue, green, and yellow. Accuracy of red-colored regions is less than 50%.

Figure 2 shows the three-dimensional structures of CS, extra-mitochondrial citrate synthase (eCS), and CS (*Thermosulfidibacter takaii* ABI70S6^T^) as predicted by the artificial intelligence system, AlphaFold2 [13,14]. In contrast to the structure of CS (Figure 2a), the structure of eCS is similar to that of CS (*Thermosulfidibacter takaii* ABI70S6^T^) (Figure 2b,c). Although the relationship between structure and function is not simple, a similar function between CS (*Thermosulfidibacter takaii* ABI70S6^T^) and eCS is predicted because of the similarity in structure. Therefore, this opens the possibility of a unique function for eCS, similar to the CS of *Thermosulfidibacter takaii* ABI70S6^T^.

Among the same cell type, individual cells have the unique ability to be multifunctional in different conditions. Technological advancements have enabled us to perform omics analyses using single cells [15]; individual expression variability has been proven in the same cell type. Functional differences in similar cell types are dependent on the cell status.

In this review, we aim to provide a comprehensive overview of the relationship between each TCA cycle-related enzyme and diseases arising from their dysfunction in the context of both mitochondrial and cytoplasmic enzymes of the TCA cycle. In addition, we discuss their possible functions and introduce the function of eCS as an example of a cytoplasmic enzyme of the TCA cycle.

## 2. TCA-Related Enzymes and Diseases Arising from Their Dysfunction

CS is the first rate-limiting enzyme in the TCA cycle. Many studies have shown that CS is essential for maintaining energy production in all cell types [16]. Although diseases caused by CS dysfunction have not yet been reported, CS knockdown in a human embryonic kidney cell line (293T) exhibited decreased ATP production and increased oxidative damage, leading to cell death in vitro [17].

In the cytosol, ATP citrate lyase (ACLY) is involved in citrate cleavage, resulting in the formation of oxaloacetate and acetyl-CoA (for fatty acid biosynthesis) [16,18]. ACLY is associated with cardiovascular abnormalities in humans; for example, ACLY deficiency in macrophages stabilizes atherosclerotic plaques in mice [19]. The atherosclerotic plaques are due to an increase in collagen deposition and fibrous cap thickness, along with a small necrotic core.

Aconitase catalyzes the reversible isomerization of citrate to isocitrate [20]. Aconitase 1 (Aco1) and aconitase 2 (Aco2), two aconitase isoforms, present in the cytoplasm and mitochondria, respectively [18]. Deficiency of Aco1 and Aco2 are associated with increased risk of optic atrophy and encephalopathy, respectively [21,22]. These diseases are caused by autosomal recessive mutations in Aco 1 and 2. Infantile cerebellar-retinal degeneration is also associated with mutations in the mitochondrial Aco2. The patients show clinical phenotypes with ophthalmological abnormalities, such as optic atrophy.

Previous studies have demonstrated that dominant defects of three TCA cycle-related enzymes, isocitrate dehydrogenase (IDH), succinate dehydrogenase (SDH), and fumarase, lead to tumor formation [6,18].

IDH associated with NADP^+^ is present as a monomer (IDH1) in the cytoplasm and as a dimer (IDH2) in the mitochondria. IDH1/2 are essential enzymes in the mitochondrial antioxidant system against oxidative stress as they synthesize nicotinamide adenine dinucleotide phosphate [1,18]. Mutation of IDH1/2 results in a decrease in α-ketoglutarate, leading to a disturbance of TCA cycle function. Dominant IDH1/2 mutations have been found in patients with gliomas and acute myeloid leukemia [6,18,23].

The SDH complex, also known as mitochondrial complex II, catalyzes the oxidation of succinate to fumarate in the TCA cycle [24]. This complex is composed of a heterotetramer of SDHA and SDHB subunits anchored to the inner mitochondrial membrane by SDHC and SDHD subunits [24]. Recently, it was found that dominant mutations in SDHB, SDHC, and SDHD cause susceptibility to paragangliomas [18,25]. SDHA mutations in germ cells have also been found in patients with paragangliomas [18,26]. In addition, SDHB mutations are related to renal cell carcinoma and T-cell acute leukemia [27,28], and SDHB, SDHC, and SDHD mutations result in gastrointestinal stromal tumors [29]. However, the reason for tumor development because of SDH mutations remains unclear.

Fumarase, in its homotetrameric active form, converts fumarate to malate [30]. The same gene encodes two isoforms, and recessive mutations of fumarase, in both the mitochondrial and cytosolic enzymes, causing severe encephalopathies [18]. In addition, dominant mutations in fumarase cause susceptibility to tumors, such as uterine leiomyomas, leiomyomatosis, renal cell cancer, ovarian cystadenomas, and breast cancer [31]. A study reported that in a patient with cancer caused by fumarase mutation, although there was a significant decrease in fumarase activity in the cells, mitochondrial fumarase was maintained at normal levels. However, no fumarase was detected in the cytosol, suggesting that cytosolic fumarase may function as a tumor suppressor [32].

**Table 1:** TCA cycle-related enzymes and diseases arising from their dysfunction.

Enzymes	Abbreviation	Localization	Diseases	References
Citrate synthase	CS	Mitochondria	Cell death (in a human cell line, in vitro)	[17]
Citrate lyase	ACLY	Cytosol	Atherosclerotic plaques (in mice, in vivo)	[19]
Extra-mitochondrial citrate synthase	eCS	Cytosol	Decrease in age-dependent male fertility (in mice, in vivo)	[33]
Aconitase	ACO1	Cytosol	Encephalopathy (in humans, in vivo)	[18,21,22]
	ACO2	Mitochondria	Optic atrophy (in humans, in vivo)	
Isocitrate dehydrogenase	IDH1	Cytosol	Gliomas, acute myeloid leukemia (in humans, in vivo)	[6,18,23]
	IDH2	Mitochondria		
Succinate dehydrogenase	SDHA	Mitochondria	Paragangliomas (in humans, in vivo)	[18,26]
	SDHB		Gastrointestinal stromal tumors, paragangliomas, renal cell carcinoma, T-cell acute leukemia (in humans, in vivo)	[18,25,27,28,29]
	SDHC		Gastrointestinal stromal tumors, paragangliomas (in humans, in vivo)	[18,25,29]
	SDHD		Gastrointestinal stromal tumors, paragangliomas (in humans, in vivo)	[18,25,29]
Fumarase (fumarase hydratase)	FH	Mitochondria	Encephalopathy, leiomyomas, leiomyomatosis, renal cell cancer, ovary cystadenomas, breast cancer (in humans, in vivo)	[18,31]
		Cytosol		
α-ketoglutarate dehydrogenase	OGDH	Mitochondria	Neurological disorder (in humans, in vivo)	[34]
Malate dehydrogenase	MDH1	Cytosol	Encephalopathy (in a human cell line, in vitro)	[35,36]
	MDH2	Mitochondria		
Malic enzyme	ME1	Cytosol	Unknown	[37,38]
	ME2	Mitochondria	Idiopathic generalized epilepsy (in humans, in vivo)	[37,39]
	ME3	Mitochondria	Unknown	[37]
Glutamate-oxaloacetate transaminase	GOT1	Cytosol	Unknown	[40]
	GOT2	Mitochondria	Neurometabolic disorder (in humans, in vivo)	[41]

One subunit of the 2-oxoglutarate dehydrogenase complex is encoded by the α-ketoglutarate dehydrogenase (OGDH) gene [34]. OGDH is located in the mitochondria and catalyzes the conversion of α-ketoglutarate to succinyl-CoA and CO_2_. Neurological disorders are caused by a decrease in the activity of OGDH and the reactive oxygen species (ROS)-induced inactivation of OGDH in brain [34].

Malate dehydrogenase (MDH) participates in the oxidation of malate to oxaloacetate. MDH 1 and MDH2 are localized in the cytosol and mitochondria, respectively [18]. In a human HEK293 cell line, MDH1 deficiency causes a metabolic disorder of the malate-aspartate shuttle, leading to severe encephalopathy with an increase in glycerol-3-phosphate [35]. Similarly, mutations in MDH2 cause early-onset severe encephalopathy in HEK293 cells because of deleterious MDH2 variants [36].

Malic enzymes (MEs) convert malate to pyruvate (the TCA carbon source) and NADPH [42]. There are three isoforms of MEs: NADP^+^-dependent malic enzyme 1 (ME1), NAD^+^-dependent malic enzyme 2 (ME2), and NADP^+^-dependent malic enzyme 3 (ME3). ME1 is present in the cytosol, whereas ME2 and ME3 are present in the mitochondria [37]. Inhibition of ME1 leads to a decrease in NADPH (functioning as an antioxidant) and an increase in ROS, and consequently, is lethal in ME2-unexpressed human gastric cancer cells [38]. Accordingly, ME1 knockdown in human gastric cancer cells suppressed tumor growth in vivo [38]. In humans, recessive ME2 mutations predispose to idiopathic generalized epilepsy because genetic variation of the ME2 gene confers susceptibility to idiopathic generalized epilepsy [39]. However, no evidence was found for disease arising from ME3 mutations.

Two isoforms of glutamate-oxaloacetate transaminase (GOT), GOT1 and GOT2, are known to be important regulators of glutamate levels [43]. GOT1 and GOT2 are localized in the cytosol and mitochondria, respectively. Although GOT1 inhibition promotes pancreatic cancer cell death, no evidence has been found for the disease [40]. As GOT2 metabolizes 5′-phosphate esters pyridoxal 5′-phosphate, a metabolically active form of vitamin B6, GOT2 deficiency causes its faulty metabolism, leading to an autosomal recessive neurometabolic disorder [41].

## 3. Ca^2+^ Signaling and Mitochondria

Calcium (Ca^2+^) is a well-known secondary messenger that regulates a variety of cellular functions, such as signal transduction, hormone secretion, cell division, and differentiation [44,45]. A decline in Ca^2+^ signals with age affects the regulation of cellular functions [46]. Ca^2+^ amplitude decreases with age [47], and leads to a decrease in ATP production. Ca^2+^ signals are important factors for neurodegenerative and aging processes [47] because alterations in Ca^2+^ signals contribute to cell death. Alterations in Ca^2+^ signals may affect metabolites and mitochondrial functions, consequently contributing to and impaired cellular function.

Intracellular free Ca^2+^ concentration is important for maintaining cell homeostasis, and is regulated by diverse molecules. Primarily, the plasma membrane Ca^2+^-ATPases function in maintaining cytoplasmic Ca^2+^ concentration by serving as a Ca^2+^ pump in the plasma membrane [48]. The sarco-endoplasmic reticulum Ca^2+^-ATPases act as Ca^2+^ pumps in the lumen of the endoplasmic reticulum (ER) [49]. The mitochondrial Ca^2+^ uniporter (MCU) regulates Ca^2+^ transport into the inner mitochondrial membrane. The increase in cytosolic Ca^2+^ determines the opening of the MCU, causing a robust increase in mitochondrial Ca^2+^ uptake. The Ca^2+^-dependent activation of TCA cycle enzymes increases the synthesis of ATP required for SERCA activity. Lack of Ca^2+^ entry causes defective oxidative metabolism in the liver, heart, skeletal muscle, and adipose tissue [50]. The electrogenic Na^+^/Ca^2+^ exchanger is related to Na^+^ influx and Ca^2+^ release across the plasma membrane [51,52].

Upon stimulation, phospholipase C (PLC) γ generally produces inositol 1,4,5-triphosphate (IP_3_). IP_3_ binds to IP_3_ receptors in the ER membrane, causing Ca^2+^ release from the ER [53]. To replenish Ca^2+^ levels in the ER, the calcium release-activated calcium channel, composed of stromal interaction molecule (STIM) and ORAI, is involved in store-operated Ca^2+^ entry [54,55,56,57]. Decreased Ca^2+^ levels inside the ER initiate the translocation of stromal interaction molecules (STIMs) on the ER membrane to interact with the plasma membrane [58]. Translocated STIMs directly interact with ORAI channels on the plasma membrane to regulate Ca^2+^ influx [58]. Additionally, in the family of transient receptor potential channels, Ca^2+^ channels in the plasma membrane, contribute to intracellular Ca^2+^ concentration in connection with store-operated Ca^2+^ entry [59,60]. Increased cytosolic Ca^2+^ concentration controls exocytosis and cellular function. Ca^2+^ signaling is critical for various biological processes because a sufficient Ca^2+^ concentration is needed to operate cellular functions, including cell proliferation and cytokine production. Remarkably, Ca^2+^ signaling in each organelle regulates organelle-specific cellular functions, such as gene regulation in the nucleus and oxidative metabolism in mitochondria [58,61]. The involvement of Ca^2+^ signaling in these processes requires the translation of Ca^2+^ concentration to cellular signals, and Ca^2+^-binding motifs are involved in such translation [58]. These motifs are common in Ca^2+^ channel proteins, proteins mediating Ca^2+^-regulated cell functions, and Ca^2+^-sensing proteins [58]. In particular, Ca^2+^-sensing proteins play an important role in transducing Ca^2+^ concentration changes to calmodulin or calcineurin, cooperatively [62].

Increases in the cytosolic Ca^2+^ concentration are also important for successful fertilization; many studies on candidate Ca^2+^ concentration-increasing factors have been reported [63,64,65,66]. Several studies provide compelling evidence that sperm contains soluble factors for the initiation of Ca^2+^ oscillations in the egg after sperm-egg fusion [67,68,69]. In frog and sea urchin eggs [70,71,72,73], cyclic adenosine dinucleotide phosphate-ribose, nicotinic acid adenine dinucleotide phosphate, cyclic guanosine monophosphate, IP_3_, and nitric oxide (NO) were identified as candidate soluble sperm factors. It has also been reported that NO can trigger Ca^2+^ release during sea urchin fertilization [72]. In mammals, the postacrosomal sheath WW domain-binding protein (PAWP) has been suggested as a factor in sperm [74]. Furthermore, Ca^2+^ oscillations in eggs were triggered by recombinant PAWP injection in clawed frogs (*Xenopus* species), cows, and pigs [75].

**Figure 3:**
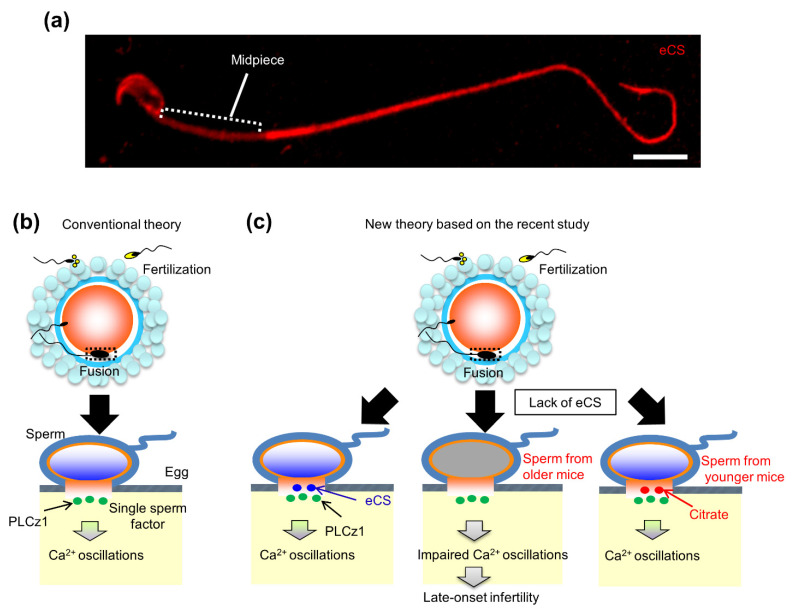
Localization and the role of eCS in triggering Ca^2+^ oscillations in the eggs. (**a**) Localization of eCS in sperm. eCS signal is totally distributed in the sperm head, midpiece (a region with helically arranged mitochondria), and tail. Especially, eCS signal is intense in the sperm head and tail. Scale bar, 1 µm. (**b**) Conventional theory. After sperm-egg fusion, the sperm-derived factors trigger Ca^2+^ oscillations in the egg. Phospholipase C zeta 1 (PLCz1) is considered to be a sperm-derived factor responsible for successful mammalian oocyte activation. (**c**) New theory based on the recent study [33]. Two sperm-derived factors, PLCz1 and eCS, are involved in triggering Ca^2+^ oscillations in the mouse egg. eCS may function to initiate Ca^2+^ oscillations, especially the first spike, alone and/or assisting PLCz1 to induce Ca^2+^ oscillations. Impressively, *eCs*-KO male mice exhibit impaired initiation of Ca^2+^ oscillations, leading to late-onset male infertility. This may be due to insufficiency of citrate synthesis by mitochondrial dysfunction with age. (**b**,**c**) are modified from source: News in National Center for Child Health and Development, Japan (https://www.ncchd.go.jp/en/news/2020/pr_20200115-e.html, accessed on 26 October 2021).

In mice, a truncated and cytosolic form of the c-kit receptor is reportedly a potential sperm factor [76]. Ca^2+^ oscillations are triggered via the activation of IP_3_ signaling, implying that PLC may be the predominant candidate factor in sperm (Figure 3b). A novel testis-specific PLC ζ was identified, and two recent studies reported that *Plcz1* KO mice are subfertile, owing to defects in triggering Ca^2+^ oscillations [77,78]. In a recent study, in which the relationship of PLCz1 with fertilization rates in infertile couples was investigated, there was no correlation between fertilization rate and PLCz1 quantity [79]. These findings raise the possibility that PLC ζ may be potential candidate factor in sperm.

In contrast, cytosolic sperm extracts have been reported to trigger an increase in Ca^2+^ concentration in newt eggs [80]; the responsible factor for this was identified as a CS [80]. A single gene produces variants corresponding to CS, and eCS lacking mitochondrial-targeting sequences (MTS) in various plant and animal species [33].

Exceptionally, the second *Cs* gene in mice encodes eCS [33]. In a recent study, eCS was found to be predominantly present in the acrosome as it lacks the MTS in mouse sperm, suggesting that eCS functions in the sperm head and not the tail containing the mid-piece (Figure 3a). In addition, an egg fused with *eCs*-KO sperm exhibited a delay in the initiation of the first spike of Ca^2+^ oscillations, despite the normal expression of PLCz1 proteins, suggesting a potential role as a sperm factor [33]. Moreover, *eCs*-KO male mice are initially fertile and exhibit declining fertility around six months after birth (corresponding to 30 years of age in humans), suggesting that eCS may play a role in age-related male infertility (Figure 3c).

Recently, it was reported that neuronal expression of eCS may regulate growth during childhood [81], as eCS expression is detected in the brain, specifically in the cerebellum and olfactory bulb. The cerebellum, which functions in social behavior, reward, and emotion, is also involved in sensorimotor processes [82]. Additionally, the olfactory bulb is a neural structure involved in olfaction [83]. In these structures, Ca^2+^ rises in response to major neurotransmitters, such as γ-aminobutyric acid and dopamine [84]. Particularly in the cerebellum, the expression of dopamine receptors and eCS is common in Purkinje cells [81,85], and it is conceivable that eCS participates in Ca^2+^ increase in response to dopamine as a Ca^2+^ inducer.

## 4. Age-Dependent eCS Function

With age, the TCA cycle as an oxidative and synthetic pathway is decreased in the jejunal epithelial cells [86]. Aconitase activity decreases with age, and consequently, the activities of other TCA cycle enzymes exhibit relatively subtle alterations. Thus, alterations in the activities of other TCA cycle enzymes lead to a decrease in the overall function of mitochondria [87]. As mentioned above, *eCs*-KO male mice are initially fertile, but fertility dropped with age (Figure 3c) [33], implying that eCS suppresses age-dependent male infertility. There could be two reasons for the age-related decline in male fertility in *eCs*-KO mice. First, a decline in mitochondrial function plays a key role in the aging process. The age-dependent decline in mitochondrial function affects subsequent CS synthesis. Therefore, eCS, which is present in extra-mitochondrial space, contributes to the fertility of aged male mice (>6-month-old).

Second, the age-related reduction in citrate content in the extra-mitochondrial space of the sperm leads to decreased male fertility. To induce Ca^2+^ oscillations after sperm-egg fusion, adequate citrate content is required. Notably, citrate content in the extra-mitochondrial region of the sperm head is more important for inducing Ca^2+^ oscillations than that in the mitochondria. It is likely that the CS and eCS ratios for citrate synthesis change with age, resulting in an age-dependent decline in mitochondrial function. Thus, eCS proteins, localized in the acrosome of the sperm, play a role in triggering Ca^2+^ oscillations, more specifically in sperm from older mice (>6-month-old).

However, testis size has been reported to be related to sperm production [88]. Although there were significant differences in testes sizes between WT and *eCs*-KO male mice, there were no significant differences in sperm function, such as motility and morphology, between the two.

## 5. Predicted Existence of Extra-Mitochondrial TCA (eTCA) Cycle

**Figure 4:**
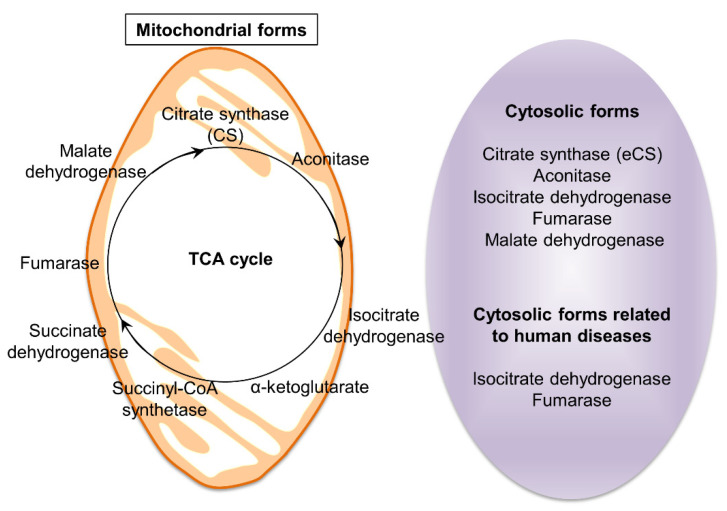
TCA cycle-related enzymes. The TCA cycle is the second stage of cellular respiration. This cycle occurs in the matrix of mitochondria and is catalyzed by eight enzymes. Interestingly, enzymes function in cytosolic forms, such as eCS, aconitase, isocitrate dehydrogenase, fumarase, malate dehydrogenase. Particularly, isocitrate dehydrogenase and fumarase were reported as human disease-related enzymes.

As depicted in Figure 1, the TCA cycle comprises eight enzymes (CS, aconitase, IDH, OGDH, succinyl-CoA synthetase, SDH, fumarase, and MDH). These enzymes are mainly distributed in the mitochondria, although most of these enzymes are also detected in the cytosolic region (Table 1 and Figure 4). Pyruvate, located at the interface between glycolysis and the TCA cycle, is an important intermediate. As mentioned above, the presence of two forms of CS, catalyzing the formation of acetyl-CoA, reinforces the importance of this interface. The mitochondrial TCA cycle has been extensively studied [89], but even after these extensive studies, the roles of cytosolic TCA cycle enzymes, including CS, are not well understood.

Aconitase catalyzes the isomerization of citrate and isocitrate. In mammals, the ACO1 (cytosolic aconitase), also known as iron regulatory protein 1 (IRP1), plays a role in sensing cellular iron homeostasis [90]. Cytosolic aconitase, upon losing an iron-sulfur cluster, becomes IRP1 [90]. Cytosolic aconitase belongs to a family of RNA-binding proteins that modulate iron metabolism in vertebrates, contributing to optimal cell growth [91].

Fumarase (also known as fumarate hydratase) is an enzyme found in both the mitochondria and the cytoplasm, and is extensively found in microorganisms, plants, and animals [92]. In mitochondria, fumarase catalyzes the reversible formation of l-malate from fumarate. In plants, cytosolic fumarase is also involved in fumarate formation [93]. Plants inhabiting cold environments are adapted to cold and freezing temperatures. Cytosolic fumarase-mediated accumulation of fumarate is essential for adaptation of *Arabidopsis thaliana* to cold [94].

Molecular cues of fumarases have been obtained from microorganisms [95]. In microorganisms, fumarases are divided into two classes, I and II, with distinct properties. Bacteria have three fumarase genes: *fumA*, *fumB*, and *fumC*. Their products, FUMA, FUMB, and FUMC, are biochemically divided into two distinct classes. Class I fumarases, FUMA and FUMB, are homologous to fumarases identified in *Euglena*. FUMA and FUMB are differentially regulated; FUMA functions in the TCA cycle, while FUMB supplies fumarate as an anaerobic electron acceptor. The class II fumarase, FUMC, is homologous to fumarases identified in *Bacillus subtilis*, *Saccharomyces cerevisiae*, and mammals. Class II fumarases are structurally conserved, with highly homologous sequences across species.

Human fumarase exists in both cytosolic and mitochondrial forms with extended N-terminus, differing only in the translation initiation site [92]; however, its role in the cytoplasm is unclear. Recent evidence has demonstrated that fumarase functions as a tumor suppressor in mammals [92]. Fumarase functions in the mitochondria, but in recent studies, it has emerged as a participant in the response to DNA double-strand breaks in the nucleus [92]. In humans, fumarase deficiency causes the formation of kidney tumors in hereditary leiomyomatosis and renal cell carcinoma (HLRCC) [31]. HLRCC is a rare genetic disease with smooth muscle growth on the skin and uterus, and is associated with a risk of developing kidney (renal) cancer. A mutation in a gene encoding fumarase is believed to cause all known cases of HLRCC. Specifically, the cytosolic form of fumarase is involved in the onset of this disease. Cytosolic fumarase plays a role in repairing DNA double-strand breaks, both through its movement from the cytoplasm to the nucleus, and enzymatic activity [96]. In other words, when fumarase is absent from cells, the DNA repair mechanism is impaired, but the administration of a high concentration of fumarate reverses this abnormality [92]. This result raises the possibility that fumarate moves actively or passively across organelles, including the mitochondria and the nucleus, and possibly other organelles.

Cytoplasmic and mitochondrial forms of isocitrate dehydrogenases (IDH1 and IDH2) dehydrate isocitrate to form oxalosuccinate [23]. Point mutations in both *IDH1* and *IDH2* have been frequently associated in the pathogenesis of a subset of gliomas, mainly low-grade gliomas and secondary glioblastomas [23]. Somatic mutations in *IDH1* cause disturbances in cell metabolism, a common feature of gliomas [23]. In addition, a novel inactivating mutation in *IDH* has been discovered in high-grade astrocytomas [97].

MDH is an enzyme that converts l-malate to oxaloacetate. In eukaryotic cells, MDH has two isoforms, MDH1 and MDH2 [42]. MDH1 is a cytoplasmic protein that transports malate into mitochondria, whereas MDH2 is a mitochondrial protein, which is part of the TCA cycle. In honeybees, three alleles encode cytosolic MDH: F, M, and S [98]. These alleles have temperature-dependent fitness benefits.

On the other hand, the cytoplasmic forms of the three enzymes, OGDH, succinyl-CoA synthetase, and SDH, remain unidentified.

Generally, ATP production using the TCA cycle occurs under aerobic conditions. Notably, cancer cells rely on glycolysis for ATP production, even under aerobic conditions [99], referred to as the Warburg effect (aerobic glycolysis) [33,34]. Although cancer cells mainly generate ATP via this process, its functions remain unclear [100]. As citrate synthesis is essential for a switch from glycolysis to the TCA cycle, dysfunction or gain-of-function of CS could be a possible cause for the Warburg effect.

Due to the lack of MTS in eCS, it is predominantly located in the sperm acrosome and not in the mitochondria. Therefore, eCS may be involved in energy production for sperm function via the eTCA cycle independently. The findings obtained from oximetric and biochemical analyses of retinal rod outer segments support the possible existence of the eTCA [101]. Additionally, *eCs*-KO male mice exhibited decreased fertility with aging (>6-month-old), suggesting an increase in eCS contribution for sperm function in older mice. This implies the possibility of the existence of the TCA cycle in the extra-mitochondrial space [101].

## 6. Clinical Trials as Metabolic Therapies

*Metabolic* therapies are a promising therapeutic strategy for the metabolic management of cancer and neurodegenerative diseases [102,103]. Metabolic control can contribute to tumor development and can be used to treat cancer [103]. Aerobic glycolysis-dependent and specific metabolic changes in cancer cells can be distinguished from normal cells. In fact, 2-deoxyglucose is used as an inhibitor of hexokinase-II to suppress tumor development in lung, breast, and prostate cancers [104]. In addition, metformin and phenformin, anti-cancer agents, are exploited as therapies for pancreatic, endometrial, and colon cancers, but these trials are still as preclinical and clinical studies [104]. Additionally, immunotherapies are pivotal in cancer therapy, and their metabolic state can regulate immune cell fate. Determining how immune cell metabolism interacts with tumor metabolism, and how this is modulated by drugs targeting metabolic enzymes might aid in the design of highly effective immunotherapies [103].

Recent reports have suggested that tumor responsiveness to chemotherapy or immunotherapy is regulated by the gut microbiota [105]. For example, treatment with butyrate, a gut microbial metabolite, directly contributes to an increase in antitumor cytotoxic CD8^+^ T cells in both mice and humans [105], suggesting that gut microbial metabolites could be effective as a part of cancer therapy. In contrast, trimethylamine-*N*-oxide and taurine are used as therapies for chronic kidney disease and diabetes [106]. The use of agents against other targets is currently in clinical trials.

To cure seizure disorders and other neurodegenerative diseases, a ketogenic diet is mainly used as metabolic therapy. The administration of ketone esters and other metabolic agents improves glucose utilization [102]. In addition, a strict ketogenic diet exhibits increased plasma ketone levels [102], suggesting that these therapies provide a multipurpose new treatment approach for a variety of disease-related neurodegeneration.

## 7. Conclusions

TCA cycle-related enzymes are essential for maintaining normal cell functions. In addition, metabolites synthesized from the TCA cycle are involved in the regulation of molecular pathways, such as apoptosis, angiogenesis, and immune system pathways. Indeed, a therapeutic approach targeting metabolic regulation has been used to treat pancreatic cancer in preclinical studies, indicating that energy metabolism is one of the tools for controlling the molecular function of cells. Metabolites are easily ingested by our bodies, although more accurate knowledge regarding their physiological functions is needed. Understanding the role of TCA cycle metabolites will elucidate their novel functions and contribute to discovering methods to overcome unsolved diseases arising from the dysfunction of TCA cycle-related enzymes.

## Data Availability

Not applicable.
